# Hepatitis E during Tocilizumab Therapy in a Patient with Rheumatoid Arthritis: Case Report and Literature Review

**DOI:** 10.1155/2018/6873276

**Published:** 2018-07-26

**Authors:** Hidekazu Ikeuchi, Kana Koinuma, Masao Nakasatomi, Toru Sakairi, Yoriaki Kaneko, Akito Maeshima, Yuichi Yamazaki, Hiroaki Okamoto, Toshihide Mimura, Satoshi Mochida, Yoshihisa Nojima, Keiju Hiromura

**Affiliations:** ^1^Department of Nephrology and Rheumatology, Gunma University Graduate School of Medicine, Maebashi, Japan; ^2^Department of Medicine and Molecular Science, Gunma University Graduate School of Medicine, Maebashi, Japan; ^3^Division of Virology, Department of Infection and Immunity, Jichi Medical University School of Medicine, Shimotsuke, Japan; ^4^Department of Rheumatology and Applied Immunology, Faculty of Medicine, Saitama Medical University, Saitama, Japan; ^5^Division of Gastroenterology and Hepatology Internal Medicine, Saitama Medical University, Saitama, Japan; ^6^Department of Rheumatology and Nephrology, Japan Red Cross Maebashi Hospital, Maebashi, Japan

## Abstract

Hepatitis E is an acute self-limiting disease caused by hepatitis E virus (HEV). Recent reports show that HEV can induce chronic hepatitis or be reactivated in immunocompromised hosts. We report a 63-year-old woman with rheumatoid arthritis (RA) who developed hepatitis E during treatment with tocilizumab. Analysis of serially stocked serum samples confirmed that hepatitis was caused by primary infection with HEV and not by viral reactivation. Her liver function improved after discontinuing tocilizumab and remained within the normal range without reactivation of HEV for >5 years after restarting tocilizumab. We also reviewed the published cases of hepatitis E that developed during RA treatment.

## 1. Introduction

Hepatic disorders are some of the most common complications that arise during rheumatoid arthritis (RA) treatment. Routine liver enzyme testing is recommended to detect the side effects of disease-modifying antirheumatic drugs (DMARDs) as well as the reactivation of viruses such as hepatitis type B (HBV), hepatitis type C (HCV), and cytomegalovirus [1, 2].

Hepatitis E is a disease of the liver that is caused by hepatitis type E virus (HEV) infection. HEV usually induces acute self-limiting hepatitis in healthy individuals. However, HEV is known to induce chronic hepatitis in immunocompromised hosts, including patients with HIV infection, chemotherapy recipients, and organ transplant recipients [3, 4]. In addition, HEV deterioration has been reported in patients after allogenic stem cell transplantation [5, 6], despite low risk of deterioration [7].

Here, we report a case of hepatitis E that developed during tocilizumab therapy for RA. In this case, we sequentially determined the serum titers of HEV antibodies and RNA before and after the onset of hepatitis using stocked serum samples. In addition, we reviewed the published cases of hepatitis E that occurred during RA treatment with DMARDs.

## 2. Case Presentation

A 63-year-old woman visited our outpatient clinic because of general malaise that lasted 6 days. She developed RA at the age of 60 years and had been treated with 400 mg monthly intravenous tocilizumab for the past 10 months and 3 mg/day prednisolone. She had no history of blood transfusion, alcohol use, travel abroad, or raw meat intake, and her joints were not tender or swollen. Disease Activity Score 28-joint count C reactive protein was 1.13. Laboratory data revealed elevated liver enzyme levels: AST, 338 IU/L; ALT, 523 IU/L; ALP, 377 IU/L; and *γ*-GTP, 68 IU/L. Blood counts, total protein, albumin, total bilirubin, electrolytes, renal tests, C reactive protein, and coagulation test results were almost within normal ranges. Her serum HBV nucleic acid levels were monitored regularly to detect HBV reactivation because she tested positive for antibodies to HBV surface and core antigens without HBs antigen before the initiation of tocilizumab. At admission, HBV DNA levels were within normal range. Tests to detect antibodies to hepatitis A and C were negative. Tests to detect antibodies to Epstein–Barr virus and cytomegalovirus were both negative for immunoglobulin M (IgM) but positive for immunoglobulin G (IgG). Abdominal ultrasound revealed normal liver morphology.

The patient was diagnosed with HEV infection (genotype 3) because tests to detect anti-HEV immunoglobulin A (IgA) antibody and HEV RNA in her sera were both positive. Tocilizumab, pregabalin, eldecalcitol, and teriparatide were discontinued, and stronger neo-minophagen C and ursodeoxycholic acid were administered. Liver enzyme levels decreased and returned to normal 3 weeks after admission, and she was discharged from our hospital. Results of HEV RNA tests were negative 6 weeks after admission. Tocilizumab and eldecalcitol were reinitiated 4 weeks after liver enzyme normalization. RA remained in remission, and liver enzymes remained stable for the subsequent 5 years under tocilizumab therapy.

Because she had been a participant in a prospective clinical study to investigate the incidence of HBV reactivation in patients receiving immunosuppressive and/or anticancer therapies [8], her sera that was collected prior to hepatitis E onset had been stored. The use of serially stocked sera for HEV detection was approved by the Ethics Committee of Gunma University Hospital (#15-61). We examined her serum for anti-HEV antibodies and HEV RNA before and after admission (Table 1). Neither anti-HEV antibodies nor HEV RNA was detected in the preadmission samples. In contrast, all of them were positive at admission. Anti-HEV IgM and IgA antibody levels peaked 1 week after admission and declined thereafter. Anti-HEV IgG antibody levels remained elevated until the final observation at 57 months. HEV RNA was detected at 0, 2, and 3 weeks after admission and was undetectable thereafter.

## 3. Discussion

HEV was previously believed to cause acute hepatitis but not chronic hepatitis. However, Kamar et al. first reported that chronic HEV infection was observed in patients after organ transplantation [3]. They further reported that HEV infection caused chronic hepatitis in >60% of solid-organ transplant recipients [9]. Chronic HEV infection was also reported in patients with HIV infection and in a patient with malignant lymphoma treated with rituximab [4, 10]. In addition, HEV persistent infection has been reported in recipients of bone marrow transplant under severe immunosuppression [5–7]. Recently, biological DMARDs (bDMARDs) or targeted synthetic DMARDs (tsDMARDs) are frequently used in RA treatment. Treatment with bDMARDs is reported to increase the risk of hepatitis B virus reactivation in patients with RA [11]. Therefore, chronic transformation of hepatitis E may occur in patients with RA who are treated with bDMARDs or tsDMARDs. In our case, sequential analysis of anti-HEV antibodies and HEV RNA using stocked serum samples clearly confirmed that our patient developed hepatitis as a result of primary acute HEV infection, but not recurrence or chronic infection of HEV. The clinical course of our patient was self-limiting, and the virus was eradicated from the serum without chronic transformation. In addition, there was neither recurrence of hepatitis nor persistent infection of HEV infection during the following 5 years, even after the reintroduction of tocilizumab.

The case reports of hepatitis E infection in patients with RA are accumulating [12–21].  Table 2 summarizes published cases of hepatitis E developed during the treatment of RA with DMARDs. Of 26 cases including ours, 20 were treated with bDMARDs or tsDMARDs with or without conventional synthetic DMARDs (csDMARDs) and 6 cases were treated with csDMARDs alone. Low-dose steroids were used in 16 cases. In most cases, DMARDs were discontinued for a certain period after the diagnosis of hepatitis. Temporal withdrawal of DMARDs may help the immune system to recover and eliminate HEV. In most patients, liver function was normalized without antiviral therapy. However, 1 patient (case 1) died of fulminant hepatitis, despite treatment with plasma exchange and continuous hemofiltration [12]. The HEV genotype detected in this patient was genotype 4, which is known to be more virulent than genotype 3. In addition, a male patient (case 25) who was administered adalimumab and methotrexate developed chronic hepatitis E [21]. The routine testing revealed elevated liver enzyme levels, and methotrexate, but not adalimumab, was discontinued. Eight months later, he was diagnosed with HEV infection, and adalimumab was discontinued. However, serum HEV RNA remained positive for more than 5 months; therefore, he was treated with ribavirin for 42 days until the test results for serum HEV RNA were negative [21].

In transplant recipients with chronic HEV infection, antiviral therapy with interferon-*α* and ribavirin as monotherapy or in combination is recommended if immunosuppressive therapy has to be continued or viral clearance is difficult to achieve even after weakening immunosuppression [22, 23]. In patients with RA who are treated with DMARDs as listed above, only 4 cases were treated with ribavirin and 3 of them had been treated with rituximab. Another patient had been treated with adalimumab before and after notification of elevated liver enzymes for several months and ultimately developed chronic hepatitis E, as described above [14, 15, 19, 21]. Based on these results, antiviral therapy may be considered only in high-risk patients: patients with HEV (genotype 4), those who had been treated with long-lasting and immunosuppressive DMARDs such as rituximab, and those with severe or chronic hepatitis. Further investigation is to clarify the adequate use of ribavirin in acute hepatitis E patients taking DMARDs.

In summary, we presented a case of RA with hepatitis E that developed during tocilizumab therapy, and we reviewed published cases of hepatitis E among patients with RA on DMARDs treatment. In RA patients whose liver dysfunction was detected at routine examination, the possibility of HEV infection should be considered. In a case of hepatitis E, discontinuation of bDMARDs or tsDMARDs is recommended, and administration of ribavirin may be necessary in high-risk patients. In addition, in our case, tocilizumab was safely used after normalization of liver function for a long term without persistent infection of HEV.

## Figures and Tables

**Table 1:** Sequential changes of liver enzymes and laboratory tests for hepatitis E.

Time after admission	AST	ALT	Anti-HEV IgG	Anti-HEV IgM	Anti-HEV IgA	HEV RNA
	(13–33 IU/L^∗^)	(6–27 IU/L^∗^)	(OD_450_ < 0.175^∗^)	(OD_450_ < 0.440^∗^)	(OD_450_ < 0.642^∗^)	(−)
−30 months	17	9	0.046	0.043	0.042	−
−20 months	16	14	0.047	0.05	0.035	−
−10 months	19	14	0.035	0.088	0.04	−
0	338	523	2.563	2.845	2.93	+
1 week	44	137	2.822	>3.000	>3.000	+
2 weeks	32	37	>3.000	2.894	2.296	+
3 weeks	22	18	2.748	2.771	1.499	+
6 weeks	27	25	2.956	2.661	1.113	−
10 weeks	22	15	>3.000	2.303	1.019	−
14 weeks	27	19	>3.000	1.863	0.799	−
22 weeks	43	28	>3.000	1.022	0.536	−
26 weeks	31	26	NA	NA	NA	NA
57 months	24	17	1.659	0.176	0.115	−

^∗^Normal range. HEV, hepatitis E virus; IgG, immunoglobulin G; IgM, immunoglobulin M; IgA, immunoglobulin A; NA, not available.

**Table 2:** Summary of cases of hepatitis E during treatment of rheumatoid arthritis.

Number	First author	Year	Reference	Sex	Age	bDMARDs/tsDMARDs	Stopping bDMARDs/tsDMARDs	csDMARDs	Stopping csDMARDs	PSL (mg/day)	HEV genotype	Ribavirin	Periods for disappearance of HEV RNA	Prognosis
1	Sugawara	2009	[12]	M	60	IFX	NA	MTX, BUC		(+)^*∗*^	4	(−)	NA	Died due to fulminant hepatitis
2	Bauer	2013	[13]	F	68	ABA	Yes	LEF	Yes	5	3	(−)	NA	Improved
3	Roux	2013	[14]	M	55	RTX	Yes	MTX	Yes	(+)^*∗*^	3	(+)	3 months	Improved
4	Bauer	2015	[15]	F	62	IFX	Yes	MTX	Yes	(−)	NA	(−)	4 weeks	Improved
5	Bauer	2015	[15]	M	72	RTX	No	MTX, LEF	Yes	(−)	NA	(−)	NA	Improved
6	Bauer	2015	[15]	F	49	TCZ	Yes	(−)	Yes	3	3f	(−)	6 weeks	Improved
7	Bauer	2015	[15]	F	69	ABA	Yes	LEF	Yes	5	3f	(−)	6 weeks	Improved
8	Bauer	2015	[15]	M	69	RTX	No	MTX	No	(−)	NA	(+)	10.5 weeks	Improved
9	Bauer	2015	[15]	M	61	RTX	No	LEF	Yes	3	NA	(−)	8 weeks	Improved
10	Bauer	2015	[15]	F	53	ABA	Yes	MTX	Yes	(−)	NA	(−)	9 weeks	Improved
11	Bauer	2015	[15]	F	44	RTX	Yes	MTX	Yes	(−)	3c	(−)	9.5 weeks	Improved
12	Bauer	2015	[15]	F	55	ETN	Yes	MTX	Yes	(−)	NA	(−)	4 weeks	Improved
13	Bauer	2015	[15]	F	60	ADA	Yes	MTX	Yes	4	3f	(−)	8 weeks	Improved
14	Bauer	2015	[15]	M	59	TCZ	Yes	MTX	Yes	7	NA	(−)	4 weeks	Improved
15	Schultze	2015	[16]	F	68	(−)		MTX	Yes	5^*∗*^^*∗*^	NA	(−)	40 days	Improved
16	Leloy	2015	[17]	F	33	TCZ	Yes	(−)		(−)	NA	(−)	NA	Improved
17	Kanda	2015	[18]	F	64	(−)		MTX, BUC	NA	(−)	3	(−)	NA	Improved
18	Kanda	2015	[18]	F	74	TOF	Yes	(−)		(+)^*∗*^	3	(−)	NA	Improved
19	Kanda	2015	[18]	F	52	(−)		MTX	NA	(−)	3	(−)	NA	Improved
20	Verhoeven	2016	[19]	F	51	RTX	Yes	(−)		(−)	NA	(+)	2 months	Improved
21	Kobayashi	2017	[20]	F	58	(−)		BUC, MIZ, ACT	No	5	NA	(−)	NA	Improved
22	Kobayashi	2017	[20]	M	61	ETN	Yes	MTX	Yes	3	NA	(−)	NA	Improved
23	Kobayashi	2017	[20]	M	67	(−)		MTX, TAC	Yes	5	NA	(−)	NA	Improved
24	Kobayashi	2017	[20]	F	52	(−)		MTX, MIZ, TAC	Yes	4	NA	(−)	NA	Improved
25	van Bijnen	2017	[21]	M	63	ADA	Yes	MTX	Yes	3	3	(+)	42 days after ribavirin	Chronic infection˗improved
26	Our case			F	63	TCZ	Yes	(−)		3	3e	(−)	6 weeks	Improved

DMARDs, disease-modifying antirheumatic drugs; bDMARDs, biologic DMARDs; csDMARDs, conventional synthetic DMARDs; PSL, prednisolone/prednisone; HEV, hepatitis E virus; IFX, infliximab; RTX, rituximab; TCZ, tocilizumab; ABA, abatacept; ETN, etanercept; TOF, tofacitinib; MTX, methotrexate; BUC, bucillamine; LEF, leflunomide; MIZ, mizoribine; ACT, actarit; TAC, tacrolimus; NA, not available. ^*∗*^Dose is not available; ^*∗*^^*∗*^weekly dosage.
